# South Asian Immigrant Men and Women and Conceptions of Partner Violence

**DOI:** 10.1007/s10903-015-0301-2

**Published:** 2015-10-15

**Authors:** F. Ahmad, J. Smylie, M. Omand, A. Cyriac, P. O’Campo

**Affiliations:** 1 Center for Research on Inner City Health, Lee Ka Shing Knowledge Institute, St Michael’s Hospital, Toronto, Canada; 2 Faculty of Health, School of Health Policy and Management, York University, 4700 Keele Street, HNES 414, Toronto, M3J 1P3 Canada; 3 Dalla Lana School of Public Health, University of Toronto, Toronto, Canada

**Keywords:** Intimate partner violence, Concept mapping, Ethnicity, South Asian, Immigrants

## Abstract

Limited knowledge exists about conceptual variations in defining intimate partner violence (IPV) by ethnicity, such as South Asian (SA) immigrant men and women. In a multi-ethnic study, we employed participatory concept mapping with three phases: brainstorming on what constitutes IPV; sorting of the brainstormed items; and interpretation of visual concept maps generated statistically. The parent study generated an overall general multi-ethnic map (GMEM) that included participant interpretations. In the current study, we generated a SA specific initial-map that was interpreted by eleven SA men and women in gender specific groups. Their interpretations are examined for similar and unique aspects across men and women and compared to GMEM. SA men and women shared similar views about sexual abuse and victim retaliation, which also aligned closely with GMEM. Both SA women and men had an expanded view of the concept of controlling behaviors compared to GMEM. SA women, unlike SA men, viewed some aggressive behaviors and acts as cultural with some GMEM congruence. SA women uniquely identified some IPV acts as private–public. We discuss implications for research and service assessments.

## Introduction

Intimate Partner Violence (IPV) is a widespread public health issue affecting all social classes and ethnicities, and impacting both women and men. The World Health Organization (WHO) defines partner violence as “any behavior within an intimate relationship that causes physical, psychological or sexual harm to those in that relationship” [1]. IPV includes acts of physical aggression, psychological abuse, forced sexual contact or other controlling behaviors. Over the last three decades significant advances have been made in assessing IPV rates, risk factors and patterns, and in evaluating treatments. However, limited understanding exists about conceptual variations in defining IPV by ethnicity for both men and women.

Ethnic diversity is on the rise due to global migration [2] and is notable in regions with a history of migration and settlement, such as Canada and the United States (US). In Canada, the ethnic mosaic has been diversifying since 1967 when immigration policy was modified, establishing a point-system based on newcomers’ skills and education and removing preferential support for Europeans. Recent cohorts of migrants to Canada include large numbers from Asia and the Middle East. In 2006, Canadians of South Asian descent became the top visible minority group [3, 4], and in 2011 they accounted for 1.5 million residents [5]. South Asia includes countries like India, Pakistan, Bangladesh, Sri Lanka and Nepal. The South Asian (SA) community is also growing quickly in the US [6].

Despite their growing numbers in North America, limited research has been conducted with the SA community with respect to IPV and its conceptualization. Some small-scale studies identify IPV as a serious issue for the community [7–10] and others report poor health outcomes for SA women with IPV experiences [11, 12]. Further, high rates of IPV are reported for women in Pakistan, Bangladesh and India with the lifetime prevalence ranging from 40 to 66 % [13–15]. There is a strong need to advance scholarly knowledge about the experiences and perspectives of the SA community in relation to IPV.

Socio-cultural norms and values are likely to influence perspectives about IPV. Only a handful of studies in North America provide such insights. Klein et al. [16] compared Whites, African Americans, Latinas and Asians in a US national sample using vignettes about couple interactions. The authors found that Asian women were the least likely, while White women were the most likely, to categorize certain interactions as domestic violence, such as neighbors having a fight involving loud screaming, or a cousin shoving and slapping his wife during dinner [16]. Yick and Agbayani-Siewart [17] found that Chinese women (n = 15) in Southern California minimized psychological aggression compared to physical or sexual IPV. Likewise, a review of literature by Srinivasan et al. [18] noted that some SA women may not recognize certain acts and behaviors as abusive due to their familial obligations and culturally prescribed roles. In 2004, a Canadian study by Ahmad et al. examined the influence of patriarchal beliefs on SA women’s own perceptions of abuse by using a vignette. This telephone-survey in Toronto with Urdu, Hindi or English speaking SA women (n = 47) found that SA immigrant women with stronger patriarchal beliefs were less likely to see spousal violence as abuse, while only 17 % regarded forced sex by a husband as a possibly violent act [19]. In 2008, Mason et al. conducted eight focus groups with Sri Lankan Tamil women in Toronto (n = 68) and found that participants defined IPV broadly to include physical, sexual, emotional/psychological and financial abuse, consistent with the WHO definition [20]. While some work is emerging, it remains unclear what kinds of acts and behaviors are perceived as abusive within each type of IPV. Lack of such knowledge leads to gaps in and poor socio-cultural sensitivity across IPV related services, problems which have been well-recognized for decades [21].

We sought to address this gap by using an innovative Concept Mapping methodology. The primary objective was to explore the perspectives of the SA community, by gender, regarding behaviors that constitute IPV. The secondary objective was to examine similarities and differences between gendered SA and multi-ethnic perspectives.

## Methods

Concept Mapping is a participant-engaged research method involving three distinct and sequential activities: *Brainstorming*, *Sorting and Rating*, and *Interpretation* [22, 23]. This is a structured process with a mix of qualitative and quantitative techniques that integrate participants’ input on a single topic of interest (i.e. perceptions on what constitutes IPV), and produces an interpretable pictorial view (i.e. map) of participant generated ideas and conceptualizations. This methodology places an emphasis on purposeful sampling in order to engage expert insights about the examined phenomenon. We provide below an overview of the larger study followed by details on the linked *Interpretation* sub-study with the SA sample. Ethical approval was obtained from the Research Ethics Board at Saint Michael's Hopsital in Toronto, Canada.

In the larger study, adult men and women from diverse ethnic and socioeconomic backgrounds were recruited if they had English language proficiency and resided in the Greater Toronto Area. Personal experience with partner violence was not a prerequisite. Specific over-sampling strategies were used to recruit self-identified ethnic minority individuals, such as members of the SA community. The recruitment strategies included placement of flyers in organizations serving the populations of interest, and snowball sampling. Interested participants called the provided phone number and eligible individuals were invited for *Brainstorming* and *Sorting and Rating* *(S&R)* activities, though retention from the first to the second activity was not mandatory. Both of these activities were available via online individual-sessions or in-person group sessions segregated by gender. In *Brainstorming* participants were asked to answer the following focal question: “What are the behaviors or attitudes that would make up the part of the relationship characterized by severe conflict, abuse, excessive control, neglect or even violence?” A total of 67 people participated (32 women, 28 men, and seven who did not specify) in the *Brainstorming* and 870 statements were collected. The research team consolidated the statements by removing duplicates and merging similar items. The final list comprises 71 statements (called “items” henceforth) and was used for the *S&R* activity. Seventy-one people (42 women and 29 men) participated in the *S&R* activity, primarily via online individual-sessions. Participants sorted the 71 items into conceptually similar groups that “made sense to them” and labeled their groups in accordance with the theme of the items. Participants rated the 71 items for importance in defining IPV and in prompting a victim to seek help; details of this component are provided in a separate article [24]. The research team entered the *Sorting* data into the Concept Mapping software that generated visual maps (more detail below). These maps formed the bases of the *Interpretation* group sessions, which were segregated by gender. The group sessions were attended by 20 multi-ethnic participants (9 women and 11 men) who were purposefully selected from the *S&R* sessions to ensure gender and ethnic representation. The multi-ethnic *Interpretation* sessions led to the creation of the General Multi-Ethnic Map (GMEM); further details of this are provided in another article [25]. All individuals provided informed written consent prior to participation in their first session. Those who continued to participate from *Brainstorming* to *S&R* and/or *Interpretation* were asked to reconfirm their consent. Participants were offered an honorarium of $40 for each activity in which they participated.

**Fig. 1:**
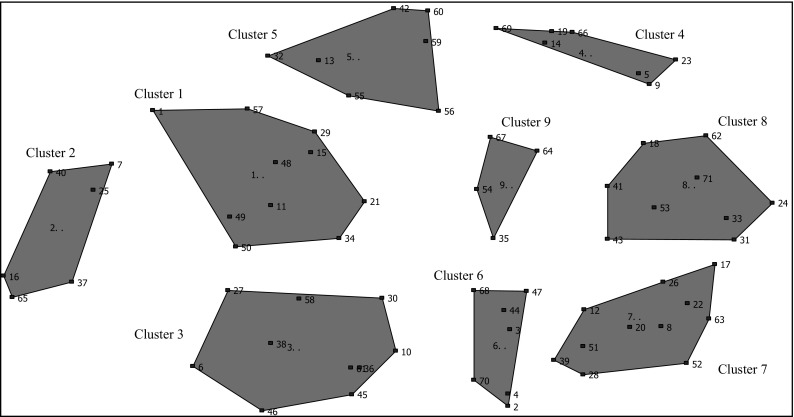
Initial concept map presented to SA participants

The reported *Interpretation* sub-study focuses on SA participants. The larger study sample included 24 SAs in the *Brainstorming* (12 women and 11 men) and 20 in the *S&R* (8 women and 12 men) activities. Using the *Sorting* SA data entered into the Concept Systems software, we first created an initial Cluster Map; this was not gender specific due to the small sample. The software employs statistical techniques of multidimensional scaling (MDS) and hierarchical cluster analysis [26]. MDS arranges points, representing items, on a spatial field based on the similarity matrix of participants’ sorted items. The results create a Point Map where items sorted together by more people appear closer to one another. The hierarchical cluster analysis then uses the means of Ward’s minimum variance method to partition the Point Map into non-overlapping clusters representing conceptual domains. From this a Cluster Map is created. The research team began with a 15 cluster solution and increased or decreased the number of clusters by one successively to identify a cluster solution where separation or merger of the clusters represented the data adequately and meaningfully. Through this review, a nine-cluster map was identified having a stress value of 0.3, which falls within the acceptable range of 0.21–0.37 provided by Kane and Trochim [23]. This SA initial-map (Fig. 1) was then presented in gender specific *Interpretation* sessions held with SA men (n = 6) and SA women (n = 5). The *Interpretation* sessions were led by gender concordant facilitators. The sessions comprised participants’ viewing of the map, ensuring all items were in the appropriate clusters, labeling of each cluster, and confirming the final number of clusters.

## Results

Eleven SA participants interpreted the SA initial map. The majority of them were first generation immigrants (i.e. ten out of eleven) who had been in Canada for ten or more years. Their ages ranged from 30 to 60 years; and all were married, all were employed, and all possessed at least a university-level education.

We report here the SA interpreted final maps by gender and compare these with the final GMEM map. The final SA maps were eight-cluster for SA men and nine-cluster for SA women. The final GMEM was a seven-cluster map. The list of cluster-content items for these final maps is presented in Table 1 for the SA and multi-ethnic subsets.

**Table 1:** South Asian (SA) and GMEM

SA women*	SA men	Location in GMEM clusters*
Psychological control (0.22)^ϕ^	Excessive control (0.26)	
35. Perpetrator keeping victim and children separated	✓	Control
34. Perpetrator controlling victim’s social contact (e.g. cannot visit friends)	✓	Control
15. Perpetrator controlling victim’s communications (e.g. emails and phone calls)	✓	Control
64. Perpetrator restricting or blocking victim’s access to education or work	✓	Control
11. Perpetrator controlling victim’s daily activities (e.g. grocery shopping, haircut …)	✓	Control
48. Perpetrator controlling victim’s important documents (e.g. passport, credit cards)	✓	Control
41. Perpetrator controlling victim’s immigration activities (e.g. deportation threat)	✓	Control
50. Perpetrator controlling and restricting family finances	✓	Psychological abuse
29. Perpetrator destroying victim’s personal property	✓	Social and Emo manipulation
13. Perpetrator interfering or blocking victim’s access to health care providers	✓	Social and Emo manipulation
57. Perpetrator imposing religious beliefs on victim and children	✓	External and cultural influences
1. Perpetrator forcing victim to work for pay	*67, 21, 18*	External and cultural influences
*49. Perpetrator denying victim or children basic necessities (e.g. clothing)*		Physical abuse
*9. Perpetrator forcibly sleep depriving victim*		Physical abuse
Victim retaliation (0.94)	Victim response (0.89)	
25. Victim provoking perpetrator to use violence	✓	Victim response to abuse
7. Victim destroying perpetrators personal property	✓	Victim response to abuse
65. Victim criticizing perpetrator	✓	Victim response to abuse
40. Victim abusing perpetrator in response to abuse	✓	Victim response to abuse
37. Victim screaming and yelling at perpetrator	✓	Victim response to abuse
16. Victim ignoring perpetrator	✓	Victim response to abuse
Emotional abuse (0.33)	Verbal abuse (0.44)	
58. Perpetrator criticizing victim (e.g. bullying, belittling, demeaning, humiliating…)	✓	Psychological abuse
36. Perpetrator making hurtful comments about physical appearance of victim	✓	Psychological abuse
61. Perpetrator cursing and name calling victim	✓	Psychological abuse
71. Perpetrator making victim cry	✓	Social and Emo manipulation
30. Perpetrator screaming and yelling at victim	✓	Social and Emo manipulation
38. Perpetrator making sexist and racist remarks about victim	✓	External and cultural influences
*12. Perpetrator making victim feel that they are crazy*	*24, 45, 6*	Psychological abuse
*54. Perpetrator maintaining a secret lifestyle and/or withholding lifestyle info from victim*		Psychological abuse
*10. Perpetrator manipulating and lying to victim*		Psychological abuse
*46. Perpetrator ignoring victim*		Psychological abuse
*47. Perpetrator abusing victim as a result of perpetrators mental illness (e.g. depression)*		Social and Emo manipulation
*31. Perpetrator abusing victim as a result of victim’s mental illness (e.g. depression)*		Social and Emo manipulation
*18. Perpetrator stalking victim*		Social and Emo manipulation
*27. Perpetrator neglecting victim when they are sick*		Physical abuse
Sexual abuse (0.46)	Sexual abuse (0.47)	
69. Perpetrator injuring victim’s breasts or genitals	✓	Sexual abuse
66. Perpetrator forcing victim into sexual acts (e.g. sodomy, view porn, oral sex)	✓	Sexual abuse
19. Perpetrator infecting victim with sexually transmitted infections	✓	Sexual abuse
5. Perpetrator punishing victim for not having sex	✓	Sexual abuse
59. Perpetrator controlling sexual activity with victim (e.g. contraception)	✓	Sexual abuse
23. Perpetrator keeping victim from enjoying sex	✓	Sexual abuse
*24. Perpetrator making unwanted sexually explicit comments to victim*	*9*	Sexual abuse
*14. Perpetrator physically abusing victim (e.g. beating, slapping, pushing, spitting)*		Physical abuse
Physical aggression (0.34)	Physical abuse (0.41)	
42. Perpetrator using a weapon to intimidate or scare victim (e.g. knife, baseball bat)	✓	Physical abuse
60. Perpetrator using a weapon to harm victim	✓	Physical abuse
56. Perpetrator controlling victim’s physical appearance (e.g. victim told what to wear)	✓	Social and Emo manipulation
*21. Perpetrator using aggressive behaviours intended to scare victim (e.g. punching wall)*	*29, 33, 49, 55, 14, 62, 32*	Social and Emo manipulation
Victim humiliation in private (0.20)	Emotional/psych abuse (0.24)	
44. Perpetrator making victim feel they can never do anything right or are ever good enough	✓	Psychological abuse
68. Perpetrator frequently becoming jealous of victim	✓	Psychological abuse
43. Perpetrator sabotaging victim’s housework (e.g. not eating home cooked meal)	✓	Psychological abuse
70. Perpetrator accusing victim of having an affair	✓	Psychological abuse
4. Perpetrator inappropriately blaming victim	✓	Psychological abuse
53. Perpetrator encouraging children to take part in violence towards victim (e.g., encouraging kids to act dismissive and demeaning toward the victim)	✓	Social and Emo manipulation
*67. Perpetrator treating victim like they are their own personal servant*	*3, 47, 12, 31, 2, 27, 46*	Social and Emo manipulation
Public humiliation (0.27)	Mental/social abuse (0.28)	
39. Perpetrator turning other people (e.g. children, family, friends) against victim	✓	Psychological abuse
22. Perpetrator publically denying any wrongdoing toward victim (e.g. in front of family/friends)	✓	Psychological abuse
28. Perpetrator allowing external parties (e.g. colleagues, extended family) to make or influence major family decisions (e.g. marriage, finances) against victim’s wishes	✓	Psychological abuse
63. Perpetrator making scenes that put down victim at social events	✓	Psychological abuse
26. Perpetrator publically humiliating victim	✓	Social and Emo manipulation
17. Perpetrator encouraging family/friends to engage in abusive behaviours/language towards victim	✓	Social and Emo manipulation
20. Perpetrator using their cultural values to excuse abuse or violence	✓	External and cultural influences
*45. Perpetrator publically disclosing details of sex life w victim to show power*	*52, 8, 53*	External and cultural influences
*51. Perpetrator emotionally blackmailing victim (e.g. threats of suicide or divorce)*		Psychological abuse
*52. Perpetrator denying to the victim any wrongdoing within their relationship*		Psychological abuse
*55. Perpetrator demonstrating public displays of power over victim (e.g. silencing, grabbing)*		Physical abuse
Cultural (0.54)	Secretive behavior/dishonesty (0.16)	
*8. Perpetrator slanting cultural, religious and moral values to encourage abuse of victim*	*54, 10*	External and cultural influences
*2. Perpetrator punishing victim on issues related to child gender (e.g. blaming women for not having boy child or forcing child gender preference)*		External and cultural influences
*32. Perpetrator preventing victim from seeing a healthcare provider of opposite gender*		External and cultural influences
*6. Perpetrator insisting on a dowry from victim or victim’s family prior to or during marriage*		External and cultural influences
Addictions (0.54)		
*3. Perpetrator abusing victim as a result of a gambling addiction*		Physical abuse
*33. Perpetrator abusing victim as a result of alcohol and/or drug use*		Physical abuse
*62. Perpetrator forcing victim to consume alcohol and/or drugs*		Physical abuse

* Items' location in clusters are compared between SA women and multi-ethnic maps

^ϕ^Stress value; Italic text refers to gender specific items

### Psychological or Excessive Control

SA women named cluster 1 of the initial map “Psychological Control” and they placed 14 items in the interpreted (or final) cluster. SA men named it “Excessive Control” with 14 items in the final cluster. There was a considerable overlap in the items placed in the final clusters by SA men and women (11/14). The final clusters for SA women and men included 100 % of the seven items contained in a cluster named “Control” in the GMEM. Notably, SA men and women included seven additional items and extended the concept of control to include financial control, forced work, imposed religious beliefs, and blocked access to health care providers.

### Emotional or Verbal Abuse

SA women named cluster 3 of the initial map “Emotional Abuse” with 14 items in the final cluster. SA men named the cluster “Verbal Abuse” with nine items. SA men and women showed a great degree of similarity in the content of this cluster and matched on shared six items. Items in the “Verbal Abuse” cluster of SA men were distributed across multiple clusters in the GMEM, unlike the SA women’s cluster. This indicates more conceptual congruence between the participant SA women and the multi-ethnic group for the emotional and psychological abuse.

### Sexual Abuse and Victim Retaliation or Response

Both SA women and men named cluster 4 of the initial map “Sexual Abuse” and placed eight and seven items in it, respectively. Six items were similar for men and women, but they differed in terms of keeping or removing item 14 on physical abuse with examples of beating, slapping, pushing or spitting, and item 24 on making unwanted sexually explicit comments. When compared with the seven-item cluster “Sexual Abuse” of the GMEM, there was a 100 % match for the SA women’s cluster (i.e. 7/7 items) and 86 % match for the SA men’s cluster (i.e. 6/7 items). Overall, high congruence existed among participants across gender and culture for the concept of sexual abuse. The cluster 2 of the initial map had six items on victim’s retaliation. SA women and men kept this cluster as it was during the map interpretation sessions and named it “Victim Retaliation” and “Victim Response”, respectively. The items in this cluster matched 100 % with a six-item cluster “Victim Response to Abuse” in the GMEM, indicating a congruent interpretation of this cluster across genders and cultures of the participants.

### Physical Aggression or Abuse

SA women named cluster 5 of the initial map “Physical Aggression” and placed four items in it. SA men named the cluster “Physical Abuse” and included 10 items. There seems to be a great degree of difference in the perspectives of SA men and women in relation to physical abuse. The SA men cluster contains three of the items that are in the SA women cluster, but is much larger, containing seven additional items. This is especially interesting because both groups had similar names for the cluster, but differed markedly with respect to the content of the cluster. Comparing these final SA clusters with a ten-item cluster “Physical Abuse” in the GMEM map showed that for the SA men’s cluster, 90 % of items matched (i.e. 9/10 items) with the GMEM cluster on “Physical Abuse”, but for the SA women’s cluster on “Physical Aggression”, just two items matched with the GMEM’s cluster “Physical Abuse”. This indicates that SA women had a distinct concept of physical aggression compared to SA men and compared to the multi-ethnic group. SA women kept item 14 on physical beating in the “Sexual Abuse” cluster.

### Private Humiliation or Emotional/Psychological Abuse

SA women named cluster six of the initial map “Victim Humiliation in Private” with seven items. SA men called this cluster “Emotional/Psychological Abuse”, keeping all initial items and adding six more. This cluster seems unique to SA women as they modified this cluster to shed light on their perspectives about behaviors which are private in nature but qualify as acts of IPV. SA men did not name any of the other clusters to reflect this perspective. In comparison to the GMEM, none of the clusters in the general map were named to reflect a focus on private or public aspects of abusive behavior.

### Public Humiliation or Mental/Social Abuse

SA women and men interpreted and labeled cluster 7 of the initial map somewhat similarly. Women named it “Public Humiliation” and placed 11 items in it. SA men named it “Mental/Social Abuse” and included 10 items. The similarity in perspectives of SA men and women is notable here. Upon comparison with the GMEM, we found that half of the items in the SA women’s cluster (i.e. 6/11 items) and the SA men’s cluster (i.e. 5/10 items) come from “Psychological Abuse” in the general map. The remaining items were distributed across different clusters in the GMEM. The concept of public or social abuse seems unique to the SA group compared with the multi-ethnic group.

### Cultural Abuse, Addiction and Secretive Behavior/Dishonesty—New Clusters

Both SA women and men dissolved cluster 8 of the initial map. Thus, the cluster-solution reduced by one cluster for SA men. However, a new cluster emerged for SA women named “Cultural” containing four items. These items were all found in a cluster called “External and Cultural Influences” containing nine items in the GMEM map. The initial map’s cluster 9 was completely dissolved by the SA women, who brought three items together to create a new cluster on “Addictions”. These items were found in the “Physical Abuse” cluster of the GMEM. SA men modified the initial cluster 9 to become “Secretive Behavior/Dishonesty”, bringing two items together from the “Psychological Abuse” cluster of the GMEM. These findings indicate cultural emphasis placed on abusive behaviors related to addiction and dishonesty by the participant SA women and men, respectively.

## Discussion

The findings generated by our exploratory study advance understanding about conceptualizations of aggressive behaviors as IPV by SA men and women. We found that the SA men and women’s conceptualizations vary compared to general multi-ethnic interpretations. To begin with, there were notable similarities across gender and ethnicity for the concepts of sexual abuse, victim retaliation and controlling behaviors, with an expansion of the controlling behavior domain by the SA group compared to the multi-ethnic group. SA men and women showed high similarity in their conceptualization of verbal/emotional abuse and did not distinguish between psychological and emotional abuse in the same way that the multi-ethnic group did. SA women were unique in their attention to the public versus the private nature of abuse. Further, the conceptualization of what comprised cultural abuse was much narrower and specific for SA women compared to the multi-ethnic group and did not emerge as an important domain for SA men. These findings are discussed in light of existing literature along with implications for further research and practice.

SA men’s and women’s perceptions of sexual abuse showed several clear differences, despite overarching congruence with each other and with the multi-ethnic sample. Previous work with SA communities also reveals certain unique perspectives [18, 19] within similarly defined general IPV domains [20]. SA women in our study included physical abuse as an act of sexual abuse. Their discussion on this item during the interpretation session demonstrated their desire to highlight gender based power imbalance as a root cause of physical abuse. While our study sample was small, several other studies point to the strong patriarchal values and rigid gender roles which normalize the subordination of women within the SA community [27–29]. Some scholars call it “three obediences” of a woman to her father, to her brother, and to her husband [30]. Because physical and sexual abuses are the types of IPV that most frequently inform research, practice and policy, the SA women’s perception of physical abuse *as a form of sexual abuse* may have important implications for the identification of IPV in this community. Further community specific research could deepen our understanding to enhance socio-cultural sensitivity of available programs and services. For instance, adaptations of interactive theater reported by Yoshihama and Tolman [31] could be offered to the SA community with nuanced concepts of sexual and physical abuse among men and women.

SA participants also expanded the conceptualization of controlling behaviors. They extended the concept by including items on financial control, forced work, imposed religious beliefs, and blocked access to health care providers. Further research could help clarify whether this distinct pattern indicates ethnic differences in the significance given to the controlling behaviors or the frequency of exposure. Analysis of the General Social Survey data of 1999 for a sample of 25–49 year old women in current marital or cohabiting relationship points towards the latter. Although not stratified by ethnicity, a higher proportion of emotional spousal abuse was found in the recent immigrant women compared to the Canadian-born women [32]. Our study provides additional insights specific to the SA community. The SA men and women in our study conceptualized verbal/emotional abuse similarly to each other but differently from the multi-ethnic group in that they did not separate psychological and emotional abuse. Perhaps this stems from a more inter-related conceptualization of mind, body and soul in Asian healing systems, as identified in other research [33]. These findings challenge the assumption of homogeneity across ethnic cultures in defining partner violence, informing scholarly debate on what constitutes IPV.

Traditionally, researchers and clinicians have focused on assessing only physical and sexual violence. For example, reports on family violence by Statistics Canada provide rates of spousal abuse by counting only incidents of physical or sexual abuse [34, 35]. Several screening tools in clinical settings ignore the measurement of emotional abuse and controlling behaviors [36]. Some studies with mainstream populations argue for the need to assess emotional abuse [37–40] but a handful of studies report the experiences and perspectives of ethnic minorities. Studies by Raj et al. [41] with SA women in Boston show that partners’ controlling acts in relation to the immigration status of women increased risk of IPV. Women in the Boston study also reported reduced sexual autonomy, increased risk of unwanted pregnancy and multiple abortions [11]. Likewise, in our study the domain clusters for emotional abuse and controlling behaviors in the SA concept maps were relatively large. These findings collectively highlight the need to assess emotional abuse and controlling aspects of couple interaction within the SA community. Asking about emotional abuse and controlling behaviors in healthcare settings could promote early detection and timely management of the risks associated with IPV. In light of delayed help-seeking reported by SA immigrant women with experiences of partner abuse, this could be particularly meaningful [42].

SA women in our study gave unique attention to the public versus the private nature of abuse, unlike SA men or the multi-ethnic group. The cultural values of familism and collectivism might have played a role in this conceptual distinction. Familism places emphasis on family relationships and, thus, matters concerning a family are considered ‘private’ [43]. Collectivism prioritizes the needs and goals of a collective (e.g. community) over an individual, and this leads to an “insider” and “outsider” group separation with a desire to protect the face [44, 45]. These cultural orientations have been previously associated with minimization of experiences of partner abuse by SA women and a delay in help-seeking [42, 46]. Our study suggests a possible link between these values and the definitions of types of abusive acts perpetrated by an intimate partner. However, it is unclear why SA participant men did not distinguish between private and public acts of partner abuse. Given that all of the SA participants who interpreted the maps were immigrants, employed and educated, it is possible that the women and the men had differing levels of attachment to these values. Others report variations in the rate of acculturation by gender and an expectation that women are often cultural ambassadors for transmitting values of the culture of origin to the next generation [47, 48]. This may explain why the SA women, unlike the men in our study, included a cluster on culture-based abuse with items pertaining to dowry, gender of newborns, difficulties in seeing a health care provider of opposite gender, and using cultural, religious and moral values to justify abusive behavior. While future research is needed to examine hypotheses generated by this study, the findings clearly highlight the need to measure and assess multiple aspects of abusive behavior for gender and ethnic inclusivity.

There were several limitations in our study. Although we used a broad recruitment strategy, it was not an easy task to recruit ethnically and socio-economically diverse participants due to the sensitive topic of research. Likewise, proficiency in the English language, required to undertake the study activities, might have introduced bias in reaching the population of interest. We could not examine the difference between the perceptions of IPV by the experience of IPV or participant socio-demographic characteristics (e.g. age, education, acculturation, income levels) due to the small sample size. The selection of articulate participants for map interpretation also limits the findings to the studied group. The sorting of the brainstormed statements might have caused participant fatigue, though we limited the number of statements. Finally, volunteer bias should warrant caution in the interpretation of the findings. Nevertheless, the concept maps generated by the SA participants and its comparison with the general multi-ethnic maps provide insights for future research and services.

In conclusion, SA men and women shared similar views about sexual abuse and victim retaliation, which also generally aligned with the views of multi-ethnic participants, although several unique aspects were identifiable. SA participants expanded the concept of controlling behaviors compared to their multi-ethnic counterparts. SA women viewed some aggressive behaviors and acts as cultural and demonstrated unique sensitivity towards the private versus public nature of abuses. Further research is needed with a larger and more diverse SA sample to examine the insights gained from our exploratory study.

## References

[1] Heise L, Garcia-Moreno C, Krug E, Dahlberg LL, Mercy JA (2002). Violence by intimate partners. World report on violence and health.

[2] Skeldon R (2013). Global migration: demographic aspects and its relevance for development.

[3] Statistics Canada. Census of population immigration, birthplace and birthplace of parents, citizenship, ethnic origin, visible minorities and aboriginal peoples. 2003. http://www.statcan.gc.ca/daily-quotidien/030121/dq030121a-eng.htm.

[4] Statistics Canada. Canada’s Ethnocultural Mosaic, 2006 Census. http://www12.statcan.ca/english/census06/analysis/ethnicorigin/toronto.cfm.

[5] Statistics Canada. 2011 national household survey: immigration, place of birth, citizenship, ethnic origin, visible minorities, language and religion. 2013. http://www.statcan.gc.ca/daily-quotidien/130508/dq130508b-eng.htm.

[6] The US Census Bureau. The American community—Asians: 2004. 2007. https://www.census.gov/content/dam/Census/library/publications/2007/acs/acs-05.pdf.

[7] Dasgupta SD (2000). Charting the course: an overview of domestic violence in the South Asian community in the United States. J Soc Distress Homeless.

[8] Ayyub R (2000). Domestic violence in the South Asian Muslim immigrant population in the United States. J Soc Distress Homeless.

[9] Raj A, Silverman JG (2002). Intimate partner violence against South-Asian women in greater Boston. J Am Med Womens Assoc.

[10] Hyman I, Mason R, Guruge S, Berman H, Kanagaratnam P, Manuel L (2011). Perceptions of factors contributing to intimate partner violence among Sri Lankan Tamil immigrant women in Canada. Health Care Women Int.

[11] Raj A, Liu R, McCleary-Sills J, Silverman JG (2005). South Asian victims of intimate partner violence more likely than non-victims to report sexual health concerns. J Immigr Health.

[12] Hurwitz EJ, Gupta J, Liu R, Silverman JG, Raj A (2006). Intimate partner violence associated with poor health outcomes in U.S. South Asian women. J Immigr Minor Health.

[13] Andersson N, Cockcroft A, Ansari U, Omer K, Ansari NM, Khan A, Chaudhry UU (2010). Barriers to disclosing and reporting violence among women in Pakistan: findings from a national household survey. J Interpers Violence.

[14] Garcia-Moreno C, Jansen HA, Heise L, Watts CH (2006). WHO multi-country domestic violence against women team. Prevalence of intimate partner violence: findings from the WHO multi-country study on women’s health and domestic violence. Lancet.

[15] Kumar S, Jeyaseelan L, Suresh S, Ahuja RC (2005). Domestic violence and its mental health correlates in Indian women. Br J Psychiatry.

[16] Klein E, Campbell JC, Soler E, Ghez M (1997). Ending the violence.

[17] Yick AG, Agbayani-Siewart P (1997). Perceptions of domestic violence in a Chinese-American community. J Interpers Violence.

[18] Srinivasan S, Ivey S, Ivey S, Kramer E, Ying Y-W (1998). Domestic violence. Immigrant women’s health.

[19] Ahmad F, Riaz S, Barata P, Stewart D (2004). Patriarchal beliefs and perceptions of abuse among South Asian immigrant women. Violence Against Women.

[20] Mason R, Hyman I, Berman H, Guruge S, Kanagaratnam P, Manuel L (2008). Violence is an international language. Tamil women’s perceptions of intimate partner violence. Violence Against Women.

[21] Campbell J, Fishwick N, Campbell J, Humphreys J (1993). Abuse of female partners. Nursing care of survivors of family violence.

[22] Trochim WMK (1989). An introduction to concept mapping for planning and evaluation. Eval Program Plan.

[23] Kane M, Trochim W (2007). Concept mapping for planning and evaluation.

[24] O’Camp P, Smylie J, Minh A, Omand M, Cyriac A (2014). Conceptualizing acts and behaviours that comprise intimate partner violence: a concept map. Health Expect.

[25] O’Campo P, Zhang YJ, Omand M, Velonis A, Yonas M, Minh A, Cyriac A, Ahmad F, Smylie J. Conceptualization of intimate partner violence: exploring gender differences using concept mapping. J Fam Violence. 2015 (accepted).

[26] The Concept System. Ithaca, NY. 2011. http://www.conceptsystems.com.

[27] Fikree FF, Pasha O (2005). Role of gender in health disparity: the South Asian context. BMJ.

[28] George U, Ramkissoon S (1998). Race, gender and class: interlocking oppressions in the lives of South Asian women in Canada. AFFLIA.

[29] Gerwal S, Bottorff JL, Hilton BA (2005). The influence of family on immigrant South Asian women’s health. J Fam Nurs.

[30] Kim IJ, Lau AS, Chang DF, Leong FTL, Inman AG, Ebreo A, Yang LH, Kinoshita L, Fu M (2006). Family violence among Asian Americans. Handbook of Asian American psychology.

[31] Yoshihama M, Tolman RM (2015). Using interactive theater to create socioculturally relevant community-based intimate partner violence prevention. Am J Community Psychol.

[32] Ahmad F, Ali M, Stewart DE (2005). Spousal-abuse among Canadian immigrant women. J Immigr Minor Health.

[33] Kleinman A (1980). Patients and healers in the context of culture : an exploration of the borderland between anthropology, medicine, and psychiatry.

[34] Statistics Canada. Family violence in Canada: A statistical profile 2000. Ottawa: Canadian Centre for Justice Statistics; 2000. Report no. 85-224-XIE.

[35] Statistics Canada. Family violence in Canada: a statistical profile 2005. Ottawa: Canadian Centre for Justice; 2005. Report no. 85-224-XIE.

[36] Coker AL, Pope BO, Smih PH, Sanderson M, Hussey JR (2001). Assessment of clinical partner violence screening tools. J Am Med Womens Assoc.

[37] Walker L (1980). The battered woman.

[38] Wagner PJ, Mongan PF (1998). Validating the concept of abuse: women’s perceptions of defining behaviors and the effects of emotional abuse on health indicators. Arch Fam Med.

[39] O’Leary KD (1999). Psychological abuse: a variable deserving critical attention in domestic violence. Violence Vict.

[40] Sackett LA, Saunders DG (1999). The impact of different forms of psychological abuse on battered women. Violence Vict.

[41] Raj A, Silverman JG, McCleary-Sills J, Liu R (2005). Immigration policies increase South Asian immigrant women’s vulnerability to intimate partner violence. J Am Med Womens Assoc.

[42] Ahmad F, Driver N, McNally MJ, Stewart DE (2009). “Why doesn’t she seek help for partner abuse?” An exploratory study with South Asian immigrant women. Soc Sci Med.

[43] Triandis HC, Marin G, Betancourt H, Lisansky J, Chang B (1982). Dimensions of familism among Hispanic and mainstream navy recruits.

[44] Dasgupta SD, Warrier S, Hegde RS (1997). In visible terms: domestic violence in the Asian Indian context.

[45] Hofstede GH (2001). Culture’s consequences: comparing values, behaviors, institutions and organizations across nations.

[46] Abraham M (2000). Speaking the unspeakable: marital violence among South Asian immigrants in the United States.

[47] Dion KK, Dion KL (2001). Gender and cultural adaptation. J Soc Issues.

[48] Dasgupta SD (1998). Gender roles and cultural continuity in the Asian Indian immigrant community in the U.S. Sex Roles.

